# Treatment of Hooping Cough

**Published:** 1852-11

**Authors:** 


*Treatment of Hooping Cough.*—In Hooping Cough, Dr. Madison, of Virginia, recommends a blister to the nucha; Dr. Golding Bird uses *conium*, in the following form: ℞ Ext. Conii, grs. xij; Alum, grs. xxv.; syrup papaver, ʒiij; aq. fœniculi ℥iij; M. A desert-spoonful every four or six hours. Dr. Spengler, in a French journal, recommends *conia* in doses of one-fortieth of a grain for children three or four months old, and one-tenth of a grain for children of as many years. He has employed it in several cases successfully.

